# Hormone, metabolic peptide, and nutrient levels in the earliest phases of rheumatoid arthritis—contribution of free fatty acids to an increased cardiovascular risk during very early disease

**DOI:** 10.1007/s10067-016-3456-x

**Published:** 2016-11-02

**Authors:** Man Wai Tang, Frieda A. Koopman, Jan P.M. Visscher, Maria J. de Hair, Danielle M. Gerlag, Paul Peter Tak

**Affiliations:** 1 Department of Clinical Immunology and Rheumatology, Amsterdam Rheumatology and Immunology Center, Academic Medical Center/University of Amsterdam, Amsterdam, The Netherlands; 2 Department of Experimental Immunology, Academic Medical Center/University of Amsterdam, Amsterdam, The Netherlands; 3 Currently also Clinical Unit Cambridge, GlaxoSmithKline, Cambridge, UK; 4 Currently also GlaxoSmithKline, Stevenage, UK; 5 University of Cambridge, Cambridge, UK; 6 Ghent University, Ghent, Belgium

**Keywords:** Autoantibodies, Biomarkers, Cardiovascular disease, Clinical trials, Metabolic disease, Rheumatoid arthritis

## Abstract

Rheumatoid arthritis (RA) is a chronic autoimmune disease associated with changes in several hormones and metabolic peptides. Crosstalk between these factors and the immune system may be important for homeostasis during inflammation. Here, we studied the levels of hormones, metabolic peptides, and nutrients in individuals at risk for developing RA (at risk). In total, 18 hormones, metabolic peptides, and nutrients were measured in fasting serum samples from 45 autoantibody-positive individuals at risk, 22 RA patients, and 16 healthy subjects. Triglyceride (TG) levels were also measured in an independent validation cohort of 32 individuals at risk, 20 early arthritis patients, and 20 healthy controls. We found an elevated TG level in individuals at risk and significantly higher TG levels in RA patients compared to healthy controls. These results were confirmed in the validation cohort. Similarly, free fatty acid (FFA) levels showed an increase in individuals at risk and were significantly higher in RA patients compared to healthy controls. In RA patients, FFA levels were positively correlated with disease activity. Pancreatic polypeptide (PP) and norepinephrine levels were highly significantly increased in individuals at risk and RA patients compared to healthy controls. TG and FFA levels are increased in RA patients and positively correlated with disease activity parameters. The results presented here suggest a role for FFAs in the pathogenesis of RA. Furthermore, PP and norepinephrine may be a biomarker that could assist in the identification of individuals at risk.

**Electronic supplementary material:**

The online version of this article (doi:10.1007/s10067-016-3456-x) contains supplementary material, which is available to authorized users.

## Introduction

Rheumatoid arthritis (RA) is a chronic autoimmune disease affecting approximately 1 % of the population worldwide. Various hormones and metabolic peptides play a role in RA, suggesting crosstalk between the endocrine system and immunity [1]. RA is associated with an increased incidence in cardiovascular morbidity and mortality [2, 3]. Previously, elevated triglyceride (TG) levels have been demonstrated in RA patients compared to healthy controls [4, 5]. TGs can be synthesized from glycerol and free fatty acids (FFAs) by a condensation reaction, and because of the relative increase in adipose tissue, FFAs are elevated in obesity [6]. An earlier study has shown an increase in FFA levels in the presence of a metabolic syndrome in established RA patients as well as in controls [7]. Previously, it has also been reported that insulin levels are higher in RA patients compared to controls [7]. This finding suggests a relationship between metabolic peptides and increased insulin resistance, which was thought to increase cardiovascular morbidity in patients with RA. Although the authors did not find a correlation with FFAs and coronary atherosclerosis, the contribution of FFAs to atherosclerosis may be independent of insulin resistance [7]. These observations suggest that apart from classical risk factors for cardiovascular disease (CVD), the secretion of metabolic peptides may also play an important role in the increased cardiovascular risk profile in RA.

It has previously been hypothesized that there may be a link between the gastrointestinal and the immune system via an interaction between cytokines and gut hormones. Peptide YY (PYY), member of the pancreatic polypeptide (PP) family, is one of the gut hormones, which could play a role in RA. The levels of PP, one of the other metabolic factors produced by the pancreas, are also increased in established RA patients compared to healthy controls [8].

In recent years, it has been recognized that the development of clinical signs and symptoms of RA is preceded by a phase characterized by systemic abnormalities including the presence of autoantibodies [9–11] and increased serum levels of C-reactive protein (CRP) [12] in the peripheral blood of individuals at risk for RA. Smoking and overweight further increase the risk of development of arthritis [13]. The underlying mechanisms are however not fully understood. To provide more insight into a possible role of hormones and metabolic peptides in the pathogenesis as well as the increased risk for cardiovascular disease in RA, we describe the hormone, peptide, and nutrient levels in a unique cohort of individuals at risk for developing RA. The levels were compared to those found in patients with established RA and healthy controls.

## Patient and methods

### Study subjects

Twenty-two patients with active RA, 45 individuals at risk for developing RA, and 16 healthy controls were included in the first part of this study. The individuals at risk for RA had arthralgia and/or a positive family history for RA, without any evidence of arthritis upon thorough physical examination. They were positive for IgM rheumatoid factor (IgM-RF) and/or anticitrullinated protein antibodies (ACPAs). These individuals at risk were characterized by the presence of systemic autoimmunity associated with RA (phase c according to [14]) with or without environmental risk factors (phase b + c according to [14]), with or without symptoms but without clinical arthritis (phase d according to [14]) and with or without genetic risk factors (phase a + b + c according to [14]). IgM-RF was measured using IgM-RF ELISA from Hycor Biomedical, Indianapolis, IN (ULN 49 IU/ml). ACPA was measured using anti-CCP2 Elisa CCPlus (Eurodiagnostica, Nijmegen, the Netherlands (ULN 25 kAU/L)).

RA patients fulfilled the 2010 ACR/EULAR classification criteria [15, 16] and had active disease defined as DAS28 >2.6 and at least one swollen joint. All RA patients were treated with non-steroidal anti-inflammatory drugs (NSAIDs) and/or disease-modifying antirheumatic drugs (DMARDs) and were biological naive. RA patients and the individuals at risk for RA were recruited via the outpatient clinic of the department of Clinical Immunology and Rheumatology of the Academic Medical Center, Amsterdam, the Netherlands. We included healthy controls, matched for gender and age, without joint complaints, history of clinical evident arthritis, and who were not known with other systemic inflammatory disease for which immunosuppressive therapy was needed; they were negative for IgM-RF and ACPA. The controls were also recruited in the Academic Medical Center, Amsterdam, the Netherlands, via advertisements.

We used another cohort to validate our findings in the test cohort in order to limit the chance on significant results by coincidence. We included an independent validation cohort, consisting of 32 individuals at risk for RA (arthralgia and elevated IgM-RF and/or ACPA serum levels without clinically manifest arthritis), 20 early arthritis patients with disease duration <6 months (*n* = 16 RA and *n* = 4 unclassified arthritis (UA)), and 20 age- and gender-matched healthy controls. Patients were classified as UA in the absence of fulfillment of disease-specific criteria, but they had elevated serum levels of RA-specific autoantibodies (IgM-RF and/or ACPA). The RA and UA patients were biologically naive.

All subjects were included at the outpatient clinic of the department of Clinical Immunology and Rheumatology of the Academic Medical Center, Amsterdam, the Netherlands. The study was performed according to the principles of the Declaration of Helsinki, approved by the institutional review board of the Academic Medical Center (2010_327#B2011135), registered in The Netherlands National Trial Register/Nederlands Trial Register (NTR2833), and all study subjects gave written informed consent.

### Study procedures

After enrollment, the individuals at risk for developing RA were followed annually for arthritis development, which was the endpoint of the study. Patients with established RA and healthy controls had one study visit. Overnight fasting blood samples were collected between 9 and 10 a.m. at baseline and again when arthritis developed in the at-risk population. Samples were directly stored on ice, centrifuged, aliquotted, and frozen at −80° within 30 min after bleeding of the subject. Catecholamines were determined in tubes containing EGTA/glutathione and glucagon using tubes containing aprotinin.

The hormones and peptides tested are described in Supplementary Table 1. Lipid levels (total cholesterol, high density lipoprotein (HDL)-cholesterol, low density lipoprotein (LDL)-cholesterol, and TG) were measured using standard laboratory techniques. All hormone measurements, except for adrenocorticotropic hormone (ACTH), cortisol, and insulin, were performed in duplicate. The mean of these duplicate means were used for the analysis. The validation cohort was used to confirm the results obtained for lipid levels.

**Table 1:** Characteristics of the study subjects

	RA (*n* = 22)	At risk for RA (*n* = 45)	Healthy control (*n* = 16)	*P* value
Age (years)	60 (46–64)	50 (41–55)	50 (43–59)	0.048
Female (*n* (%))	15 (68)	32 (71)	9 (56)	0.58
BMI (kg/m^2^)	25.0 (21.7–27.8)	25.8 (23.4–30.1)	23.8 (22.5–25.7)	0.20
Alcohol use (*n* (%))	11 (50)	22 (49)	13 (81)	0.08
Current smoker (*n* (%))	4 (18)	14 (31)	1 (6)	0.14
Cigarettes/day^a^	18 (13–25)	10 (5–15)	20 (20–20)	0.12
Pack years^a^	40.5 (27.5–50.5)	12.5 (10.0–25.0)	34 (34.0–34.0)	0.09
IgM-RF positive (*n* (%))	19 (86)	32 (71)	0 (0)	NA
IgM-RF titer (kU/l)^b^	277 (144–360)	126 (89–261)	NA	NA
ACPA positive (*n* (%))	17 (77)	29 (64)	0 (0)	NA
ACPA titer (kAU/L)^b^	379 (104–982)	375 (109–1003)	NA	NA
Disease duration (months)	10 (2–85)	NA	NA	NA
VAS GDA (mm)	47 (27–64)	31 (15–54)	0 (0–0)	NA
TJC28 (*n*)	5 (4–8)	1 (0–3)	0 (0–0)	NA
SJC28 (*n*)	2 (2–5)	0 (0–0)	0 (0–0)	NA
ESR (mm/h)	15 (8–27)	5 (2–9)	2 (2–5)	NA
CRP (mg/l)	4.6 (2.0–10.3)	1.4 (0.8–4.0)	1.4 (0.6–3.5)	NA
DAS28	4.32 (3.69–4.82)	NA	NA	NA
NSAIDs (*n* (%))	10 (46)	15 (33)	0 (0)	NA
Corticosteroids (*n* (%))	6 (27)	0 (0)	0 (0)	NA
Methotrexate (*n* (%))	17 (77)	0 (0)	0 (0)	NA
HCQ (*n* (%))	3 (14)	0 (0)	0 (0)	NA

Data presented as median (interquartile range) or number (percentage). *P* values for Kruskal-Wallis test for continuous variables and Pearson chi-square test for categorical variables. *P* < 0.05 is considered statistically significant

*IgM-RF* IgM rheumatoid factor, *RA* rheumatoid arthritis, *BMI* body mass index, *ACPA* anticitrullinated protein antibodies, *VAS GDA* patient visual analog scale (range 0–100 mm) global disease activity, *TJC28* tender joint count of 28 joints, *SJC28* swollen joint count of 28 joints, *ESR* erythrocyte sedimentation rate, *CRP* C-reactive protein, *HCQ* hydroxychloroquine, *NA* not applicable

^a^Only in smokers

^b^Only in positive patients or individuals

The following clinical and disease activity parameters were obtained during the study visits: age, sex, body mass index (BMI), alcohol use, smoking status, IgM-RF and ACPA status, disease duration, use of NSAIDs, and/or DMARDs. Patient visual analog scale for global disease activity (VAS GDA) of 0–100 mm, tender joint count of 28 joints (TJC28), swollen joint count of 28 joints (SJC28), erythrocyte sedimentation rate (ESR; in mm/h), C-reactive protein (CRP; mg/L), and disease activity score evaluated in 28 joints (DAS28) were also measured. In individuals who developed arthritis, an additional visit was performed and all clinical and disease activity parameters mentioned above were assessed, including a 68 joint score to confirm the presence of arthritis.

### Statistical analysis

Data are presented as median (IQR) unless otherwise indicated. Differences between study groups were analyzed using Kruskal-Wallis or Mann-Whitney U test where appropriate. We considered a *p* value of <0.05 as significant in this exploratory study. The differences found in this exploratory study should be evaluated in future studies. Categorical data were analyzed using chi-square test or, if more appropriate, Fisher’s exact test. Correlations between variables were analyzed using Spearman’s rank correlation coefficient. Statistical analysis was performed using IBM SPSS Statistics 18 (SPSS Inc., Chicago, IL).

## Results

The characteristics of the study subjects in the first part of this study are summarized in Table 1. Three of the 45 individuals at risk for developing RA (referred to as “at-risk” individuals) (7 %) developed RA during follow up after 18 (16–22) months. The RA patients were significantly older compared to at-risk individuals and healthy controls. Healthy controls were matched for age with the at-risk individuals rather than the RA patients, because on average age is higher in RA patients than in at-risk individuals. Supplementary Table 2 shows the characteristics of the study subjects in the independent validation cohort. From the 32 at-risk individuals, 10 subjects developed arthritis and 22 individuals did not develop arthritis after 5 years of follow up.

**Table 2:** Endocrine hormone and metabolic peptide levels in at-risk individuals, patients with RA, and healthy controls

	RA (*n* = 22)	Healthy controls (*n* = 16)	*P* value^b^	At risk for RA (*n* = 45)	*P* value^c^
Catecholamines					
Epinephrine (nmol/L)	0.17 (0.03–0.34)	0.15 (0.06–0.18)	0.47	0.17 (0.06–0.26)	0.64
NE (nmol/L)	2.45 (1.51–3.26)	2.15 (1.49–2.71)	0.41	3.02 (2.24–4.15)	0.017
HPA axis response					
ACTH (ng/L)^a^	19.3 (12.1–26.9)	18.7 (13.4–23.6)	0.97	19.2 (12.7–27.5)	0.99
Cortisol (nmol/L)^a^	349 (246–457)	319 (288–366)	0.87	323 (222–422)	0.96
IL-6 (pg/ml)	27.7 (9.3–80.9)	7.5 (0.9–66.7)	0.22	25.1 (3.8–54.5)	0.23
Cortisol/IL-6 ratio^a^	13.8 (3.0–16.9)	16.7 (3.2–93.0)	0.48	11.4 (5.3–35.1)	0.80
Sex hormones					
Estradiol (nmol/L)	0.03 (0.02–0.08)	0.05 (0.03–0.09)	0.23	0.04 (0.02–0.08)	0.52
FSH (IU/L)	15.1 (7.2–49.7)	6.4 (3.8–57.3)	0.30	8.0 (3.2–71.7)	0.60
LH (IU/L)	12.4 (4.9–26.2)	3.9 (3.0–27.7)	0.28	7.1 (3.9–33.6)	0.45
Prolactin (μg/L)	9 (7–17)	10 (7–12)	0.73	9 (7–13)	0.95
Other hormones					
GH (mU/L)	2.10 (1.00–2.90)	1.35 (0.20–3.00)	0.17	1.1 (0.50–3.20)	0.22
TSH (mU/L)	1.85 (1.20–2.70)	1.90 (1.60–2.15)	0.80	1.55 (1.25–2.50)	0.51
Peptides					
TGs (mmol/L)	1.03 (0.75–1.29)	0.70 (0.59–1.02)	0.036	0.94 (0.72–1.15)	0.11
FFAs (mmol/L)	0.59 (0.47–0.65)	0.40 (0.35–0.50)	0.011	0.53 (0.40–0.59)	0.024
Glucagon (ng/L)	87 (75–107)	82 (68–94)	0.15	89 (74–99)	0.34
Glucose (mmol/L)	5.1 (4.8–5.4)	5.2 (4.8–5.4)	0.89	5.2 (4.8–5.4)	0.77
Insulin (pmol/L)	78 (41–94)	54 (32–62)	0.13	57 (43–100)	0.32
PP (pmol/L)	34 (23–58)	10 (6–27)	0.004	31 (21–45)	0.003

Data presented as median (interquartile range). *P*# values for Mann-Whitney U test (between RA patients and healthy controls) and *P*## values for Kruskal-Wallis test (between all the three groups). *P* value <0.05 or after Bonferroni correction <0.0028 is considered statistically significant after Bonferroni correction

*RA* rheumatoid arthritis, *NE* norepinephrine, *HPA* axis response hypothalamic-pituitary-adrenal axis response, *ACTH* adrenocorticotropic hormone, *IL-6* interleukin 6, *FSH* follicle stimulating hormone, *LH* luteinizing hormone; GH growth hormone; *TSH* thyroid stimulating hormone, *TG* triglycerides, *FFAs* free fatty acids, *PP* pancreatic polypeptide

^a^Six RA patients using corticosteroids were excluded from the analysis

^b^ *P* values for Mann-Whitney U test (between RA patients and healthy controls)

^c^ *P* values for Kruskal-Wallis test (between all the three groups)

### Most hormone levels are comparable in at-risk individuals, patients with RA and healthy controls

**Fig. 1:**
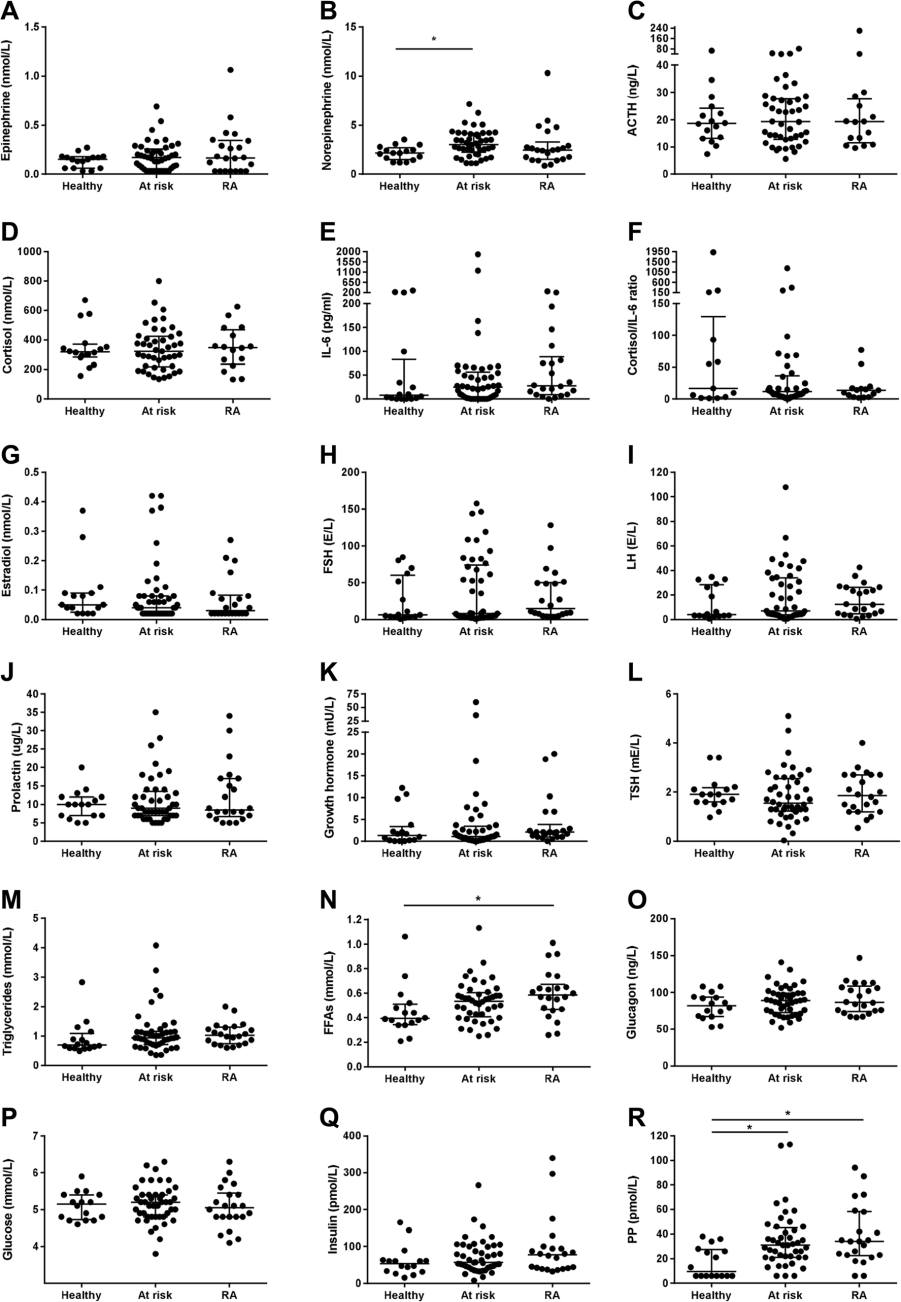
Hormones and peptides in healthy controls, at-risk individuals, and patients with RA. The serum **a** epinephrine, **b** norepinephrine, **c** adrenocorticotropic hormone (ACTH), **d** cortisol, **e** IL-6, **f** cortisol/IL-6 ratio, **g** estradiol, **h** follicle stimulating hormone (FSH), **i** luteinizing hormone (LH), (**j**) prolactin (PRL), **k** growth hormone (GH), **l** thyroid stimulating hormone (TSH), **m** triglycerides (TG), **n** free fatty acids (FFAs), **o** glucagon, **p** glucose, **q** insulin, and **r** pancreatic polypeptide (PP) level in healthy individuals, at-risk individuals, and patients with RA. *One dot* represents an individual and the median (interquartile range) is plotted as a *line* in the middle. Kruskal-Wallis test was used to detect differences in hormone levels between the groups. * *P* < 0.05028 (after Bonferroni correction)

Figure 1 and Table 2 show the hormone levels and peptide levels in at-risk individuals, patients with RA, and healthy controls. There were significantly higher norepinephrine levels in at-risk individuals (3.02 (2.24–4.15) nmol/L) compared to RA patients (2.45 (1.51–3.26) nmol/L) and healthy controls (2.15 (1.49–2.71) nmol/L; *P* = 0.017). The norepinephrine levels did not correlate with clinical and laboratory disease parameters in either RA patients or in at-risk individuals. The epinephrine levels were not different between the three groups. The levels of ACTH, cortisol, and cortisol/IL-6 ratio were also on average comparable between the three groups. IL-6 levels were higher in both individuals at risk (25.1 (3.8–54.5) pg/ml) and RA patients (27.7 (9.3–80.9) pg/ml) compared to healthy controls (7.5 (0.9–66.7) pg/ml; *P* = 0.23). The sex hormones estradiol, follicle stimulating hormone (FSH), and luteinizing hormone (LH) were comparable between the three groups. PRL levels were also similar in the at-risk individuals and RA patients compared with healthy controls. Similarly, the neuroendocrine hormones growth hormone (GH) and thyroid stimulating hormone (TSH) were comparable between the groups.

### FFAs are elevated in at-risk individuals and correlated with disease activity parameters

**Fig. 2:**
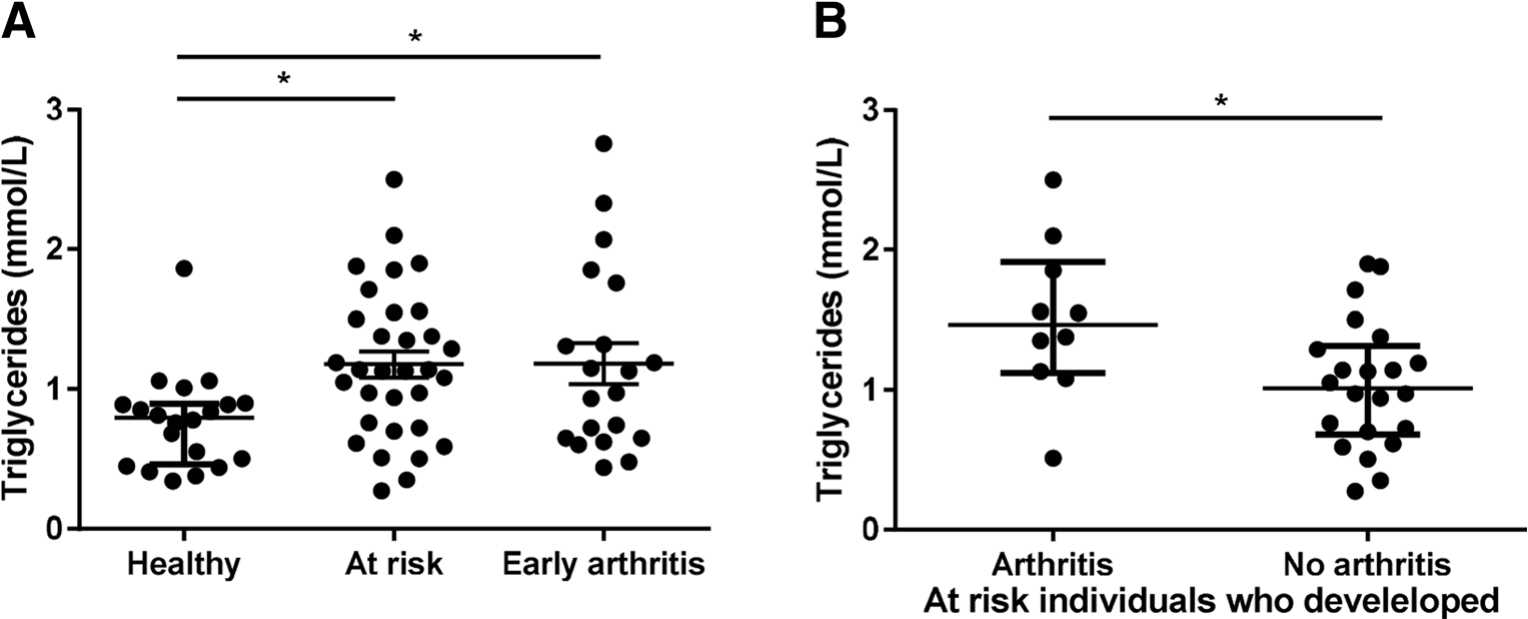
The TG levels in healthy controls, at-risk individuals, and patients with early arthritis (validation cohort). **a** The serum TG levels were higher in at-risk individuals and patients with early arthritis compared to healthy controls. **b** The TG levels were significantly higher in at-risk individuals who developed arthritis compared to individuals who did not develop arthritis. *One dot* represents an individual and the median (interquartile range) is plotted as a *line* in the middle. Kruskal-Wallis or Mann-Whitney U test was used to detect differences in hormone levels between the groups. *P < 0.05

In the test cohort, we found higher TG levels in at-risk individuals and significantly higher TG levels in RA patients compared to healthy controls (0.94 (0.72–1.15) mmol/L, 1.03 (0.75–1.29) mmol/L, and 0.70 (0.59–1.02) mmol/L, respectively; *P* = 0.11). We could confirm these findings in the independent validation cohort and found significantly increased TG levels in 20 early arthritis patients (consisting of RA and UA patients) compared to 20 healthy controls matched for age and gender (1.05 (0.65–1.54) mmol/L and 0.80 (0.48–0.90), respectively; *P* = 0.036 (Fig. 2). Moreover, in this validation cohort of 32 at-risk individuals, we observed significantly higher TG levels in baseline serum of 10 individuals who developed arthritis during follow up (1.47 (1.12–1.91) mmol/L) compared to 22 individuals who did not develop arthritis (1.01 (0.68–1.31) mmol/L) (*P* = 0.030).

**Fig. 3:**
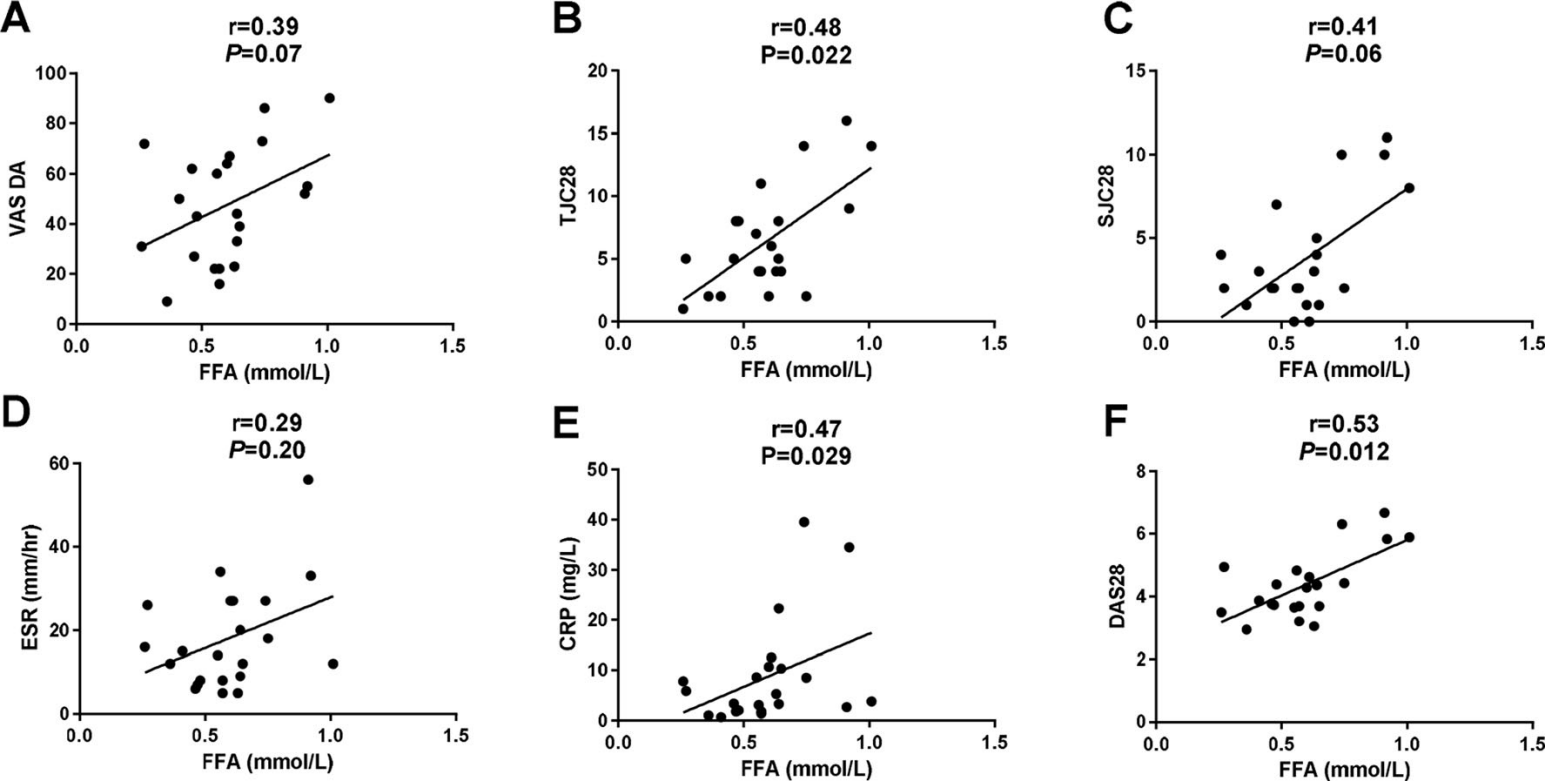
FFA level correlated moderately with several clinical and laboratory disease parameters in the RA patients. The correlations are shown between FFAs and **a** visual analog scale for global disease activity (VAS DA), **b** tender joint count 28 (TJC28), **c** swollen joint count 28 (SJC28), **d** erythrocyte sedimentation rate (ESR, mm/Hr), **e** C-reactive protein (CRP, mg/L) and disease activity score 28 (DAS28). *One dot* represents an individual. Spearman’s rank-order correlation coefficients were used to assess the correlations. * *P* < 0.05

In line with this, we found a trend toward increased FFA levels in the at-risk individuals (0.53 (0.40–0.59) mmol/L) and significantly higher levels in RA patients (0.59 (0.47–0.65) mmol/L) compared to healthy controls (0.40 (0.35–0.50) mmol/L; *P* = 0.024), independent of gender. The association of the FFA levels in the three different groups was not confounded by age, BMI, current smoking, or other variables measured (using regression analysis). Using the reference upper limit of 0.44 mmol/L as cutoff, 30 (67 %) at-risk individuals had elevated FFA levels compared to 18 (82 %) RA patients and 5 (31 %) healthy controls (*P* = 0.005). Interestingly, the FFA levels correlated in RA patients with TJC28 (*r* = 0.48 and *P* = 0.022), CRP (*r* = 0.47 and *P* = 0.029), and DAS28 (*r* = 0.53 and *P* = 0.012) and a trend toward a positive correlation with VAS GDA (*r* = 0.39 and *P* = 0.07) and SJC28 (*r* = 0.41 and *P* = 0.06) was seen (Fig. 3). In the at-risk individuals without arthritis, we did not observe a correlation between FFA levels and clinical and laboratory disease parameters. The PP levels were significantly higher both in at-risk individuals (31 (21–45) pmol/L) and RA patients (34 (23–58) pmol/L) compared to healthy controls (10 (6–27) pmol/L); *P* = 0.003. The PP levels did not correlate with clinical and laboratory disease parameters in either RA patients or in at-risk individuals.

Because of the small number of the at-risk individuals developing arthritis in this study, no conclusions can be drawn on the role of these hormones and peptides in the risk of developing arthritis. The values for these individuals are described in Table 3.

**Table 3:** Baseline hormone/peptide levels in individuals developing arthritis

	At risk for RA (*n* = 45) baseline	*At risk who did not developed arthritis* (*n = 42*) *baseline*	*At risk who developed arthritis* (*n = 3*) *baseline*	*P* value	*At risk who developed arthritis* (*n = 2*) *arthritis visit*
Catecholamines					
Epinephrine (nmol/L)	0.17 (0.06–0.26)	*0.17* (*0.06–0.27*)	*0.13* (*0.03–0.19*)	*0.42*	*0.08* (*0.03–0.12*)
NE (nmol/L)	3.02 (2.24–4.15)	*2.96* (*2.42–4.20*)	*3.14* (*1.70–3.50*)	*0.78*	*2.24* (*1.85–2.62*)
HPA axis response					
ACTH (ng/L)^a^	19.2 (12.7–27.5)	*19.3* (*12.7–27.5*)	*13.1* (*9.3–36.4*)	*0.65*	*23.7* (*13.7–33.6*)
Cortisol (nmol/L)^a^	323 (222–422)	*341* (*222–428*)	*279* (*135–287*)	*0.12*	*304* (*270–337*)
IL-6 (pg/ml)	25.1 (3.8–54.5)	*25.5* (*2.1–54.5*)	*22.6* (*18.1–163.3*)	*0.49*	*60.8* (*30.6–91.0*)
Cortisol/IL-6 ratio^a^	11.4 (5.3–35.1)	*12.1* (*5.3–40.9*)	*7.4* (*1.7–12.7*)	*0.34*	*7.0* (*3.0–11.0*)
Sex hormones					
Estradiol (nmol/L)	0.04 (0.02–0.08)	*0.04* (*0.02–0.08*)	*0.10* (*0.02–0.42*)	*0.32*	*0.16* (*0.08–0.24*)
FSH (IU/L)	8.0 (3.2–71.7)	*8.1* (*3.2–71.7*)	*4.4* (*1.6–107.9*)	*0.62*	*3.4* (*3.0–3.8*)
LH (IU/L)	7.1 (3.9–33.6)	*7.8* (*3.8–33.6*)	*4.3* (*3.9–47.6*)	*0.95*	*4.6* (*3.3–5.9*)
Prolactin (μg/L)	9 (7–13)	*9* (*7–14*)	*8* (*7–10*)	*0.63*	*9* (*7–11*)
Other hormones					
GH (mU/L)	1.1 (0.50–3.20)	*1.05* (*0.30–3.70*)	*1.30* (*1.10–2.20*)	*0.65*	*1.50* (*1.40–1.60*)
TSH (mU/L)	1.55 (1.25–2.50)	*1.60* (*1.30–2.40*)	*0.70* (*0.59–2.60*)	*0.23*	*1.13* (*0.55–1.70*)
Peptides					
TGs (mmol/L)	0.94 (0.72–1.15)	*0.94* (*0.71–1.15*)	*1.01* (*0.90–1.38*)	*0.54*	*1.02* (*0.78–1.25*)
FFAs (mmol/L)	0.53 (0.40–0.59)	*0.53* (*0.40–0.61*)	*0.53* (*0.37–0.55*)	*0.57*	*0.45* (*0.42–0.49*)
Glucagon (ng/L)	89 (74–99)	*89* (*71–99*)	*89* (*76–95*)	*0.89*	*97* (*95–99*)
Glucose (mmol/L)	5.2 (4.8–5.4)	*5.2* (*4.8–5.4*)	*5.2* (*5.0–5.8*)	*0.51*	*5.1* (*5.0–5.5*)
Insulin (pmol/L)	57 (43–100)	*60* (*43–103*)	*57* (*35–73*)	*0.60*	*50* (*26–73*)
PP (pmol/L)	31 (21–45)	*31* (*21–41*)	*65* (*6–112*)	*0.42*	*43* (*6–80*)

Data presented as median (interquartile range). *P* values for Mann-Whitney U test for continuous variables. *P* < 0.05 is considered statistically significant

*RA* rheumatoid arthritis, *NE* norepinephrine, *HPA axis response* hypothalamic-pituitary-adrenal axis response, *ACTH* adrenocorticotropic hormone, *IL-6* interleukin 6, *FSH* follicle stimulating hormone, *LH* luteinizing hormone, *GH* growth hormone, *TSH* thyroid stimulating hormone, *TG* triglycerides, *FFAs* free fatty acids, *PP* pancreatic polypeptide. * 6 RA patients using corticosteroids were excluded from the analysis

## Discussion

The results presented here show the levels of hormones and metabolic peptides in a cohort of individuals at risk of developing RA, patients with established RA, and healthy controls. We confirmed that TG and FFA levels are elevated in established RA, and we found that the FFA levels correlate with disease activity parameters. Of importance, we also found a clear trend toward elevated TG and FFA levels in individuals at risk for RA. Thus, the changes in lipid profile appear to precede the development of clinically manifest RA. There were significantly higher TG levels in baseline serum of subjects who developed arthritis during follow up compared to those who did not. PP levels were also highly significantly increased in both individuals at risk for RA and established RA patients. Although it has previously been suggested that the balance between hormones and metabolic peptides on the one hand and proinflammatory and anti-inflammatory cytokines on the other could play an important role in inflammation [1], most other hormones and peptides were comparable between the three study groups described here.

Previous studies have shown elevated levels of TG in established RA patients [4, 5]. TG levels correlated positively with ESR in one study [17], although others could not confirm this [7]. In our study, correlations between TG levels and clinical and laboratory disease parameters were found neither in RA patients nor in at-risk individuals. Since TGs can be synthesized from FFAs and hydrolysis of TGs will release FFAs, it was interesting to measure the FFAs as well. In line with the findings for TGs, a trend toward increased FFAs in at-risk individuals and significantly higher levels in RA patients compared to healthy controls were observed. Moreover, FFAs were positively correlated with several clinical and laboratory disease parameters in RA patients. The increased FFA levels in established RA are in line with a previous report, which also showed a positive correlation between FFA and CRP levels. In that study, FFA levels were higher in RA patients in the presence of the metabolic syndrome [7]. The trend toward increased FFA levels during the preclinical stage of RA described here is of interest in light of another study in which we showed that obesity is an independent risk factor for the development of RA in subjects at risk of developing this disease based on their autoantibody profile [13]. In an overweight or obese state, the adipose tissue and even the number of adipocytes are relatively increased. FFAs are released from adipocytes by lipolysis of TGs [6]. Recently, it has been suggested that FFAs could directly contribute to articular inflammation and degradation in inflammatory joint diseases. In RA synovial fibroblasts, FFAs dose-dependently enhanced the secretion of IL-6, IL-8, and monocyte chemoattractant protein 1 (MCP-1), as well as matrix metalloproteinase 1 (MMP1) and matrix metalloproteinase 3 (MMP3) [18]. These data suggest a possible role for FFAs in the pathogenesis of RA, and it can be hypothesized that the increased cardiovascular risk in the development of arthritis might be partly explained by elevated production of FFAs in obese RA patients.

The sympathetic nervous system may have an anti-inflammatory effect during inflammation via the secretion of catecholamines [19, 20]. The norepinephrine levels were significantly higher in at-risk individuals, and there was a trend toward higher levels in established RA patients compared to healthy individuals, while epinephrine levels were similar between the groups. This may represent an attempt to control inflammation.

Consistent with previous literature showing that basal peripheral blood levels of ACTH and cortisol are not different between patients with chronic inflammatory diseases and healthy controls [21, 22], we found similar levels of ACTH and cortisol between the three groups. The hypothalamic-pituitary-adrenal (HPA) axis response is relatively inadequate in RA, which is reflected by a low cortisol/IL-6 ratio [23]. In line with these findings, we demonstrated a trend toward lower cortisol/IL-6 ratios not only in established RA but also in subjects at risk of developing RA.

Interestingly, the PP levels were significantly higher in both at-risk individuals and RA patients compared to healthy controls, although the PP levels did not correlate with clinical disease parameters. The findings in established RA are in line with previous work [8]. It has previously been hypothesized that there may be a link between the gastrointestinal endocrine axis and the immune system via an interaction between proinflammatory cytokines and gut hormones. PYY, member of the PP family, is one of the gut hormones, which could play a (minor) role in RA [24]. It is conceivable that PP is more important in the earliest phase of RA.

A limitation of this study is the relatively small sample size. Of importance, however, the key results could be confirmed in an independent cohort. A potential caveat in studies of hormone and metabolic peptide levels is that they may vary based on day-night rhythm, fasting versus non-fasting state and gender. To address this, we have used fasting serum samples, obtained at the same time in the morning and corrected for gender in the analysis.

To our knowledge, this is the first study in which hormones and metabolic peptides have been determined systemically in a cohort of at-risk individuals, patients with RA, and healthy controls in one study. Here, we show that especially FFA levels are elevated in RA and correlate with disease activity. The data support the notion that FFAs play an important role in the pathogenesis of RA in which they may contribute to the increased risk of cardiovascular diseases. Further research of FFAs might lead to the identification of new therapeutic targets for the treatment and prevention of RA [25]. Furthermore, norepinephrine and PP might be a new biomarker that may help to improve prediction models in individuals at risk of developing RA.

## Electronic supplementary material

ESM 1 (DOC 49 kb).

ESM 2 (DOC 41 kb).

## Abbreviations

ACPA: Anti-citrullinated protein antibodies
ACTH: Adrenocorticotropic hormone
BMI: Body mass index
CRP: C-reactive protein
CVD: Cardiovascular disease
DAS28: Disease activity score evaluated in 28 joints
DMARDs: Disease-modifying antirheumatic drugs
ESR: Erythrocyte sedimentation rate
FSH: Follicle stimulating hormone
GDA: Global disease activity
GH: Growth hormone
HDL: High-density lipoprotein
HPA: Hypothalamic-pituitary-adrenal
IGM-RF: IgM rheumatoid factor
LDL: Low-density lipoprotein
LH: Luteinizing hormone
MCP-1: Monocyte chemoattractant protein 1
MMP-1: Matrix metalloproteinase 1
MMP-3: Matrix metalloproteinase 3
NSAIDs: Non-steroidal anti-inflammatory drugs
PP: Pancreatic polypeptide
PYY: Peptide YY
RA: Rheumatoid arthritis
SJC28: Swollen joint count of 28 joints
TG: Triglyceride
TJC28: Tender joint count of 28 joints
TSH: Thyroid stimulating hormone
UA: Unclassified arthritis
VAS: Visual analogue scale
