# Supplementary material for: Hormone, metabolic peptide, and nutrient levels in the earliest phases of rheumatoid arthritis—contribution of free fatty acids to an increased cardiovascular risk during very early disease
Source: Clin Rheumatol. 2016 Nov 2;36(2):269–78. doi: 10.1007/s10067-016-3456-x (PMC5290053; doi:10.1007/s10067-016-3456-x)
Supplement: Supplementary file 1 — (DOC 49 kb). [file 10067_2016_3456_MOESM1_ESM.doc]

**Supplementary Table 1. Neuroendocrine hormone and metabolic peptide assays**

|  | **Type of assay (manufacturer)** | **Manufacturer** | **Intraassay coefficient of variation** | **Interassay coefficient of variation** | **Total coefficient of variation** | **Detection limit** | **Reference value** |
| --- | --- | --- | --- | --- | --- | --- | --- |
| **Catecholamines** | | | | | | | |
| (Nor)  epinephrine | in-house high performance liquid chromatographic (HPLC) | Norepinephrine and epinephrine were selectively isolated by liquid-liquid extraction and derivatized to fluorescent components with 1,2-diphenylethylenediamine. The fluorescent derivatives were separated by reversed phase liquid chromatography and detected by scanning fluorescence detection. | Norepinephrine 6–8%  Epinephrine  6-8% | Norepinephrine 7–12%  Epinephrine  7-10% |  | Both 0.05 nmol/L | <0.55 |
| **HPA axis response** | | | | | | |  |
| ACTH | luminescence enzyme immunoassay | Immulite 2000, Siemens Healthcare Diagnostics B.V., Breda, The Netherlands | 3.0–8.9% | 5.2–8.9% |  | 1 ng/L | <1-55 |
| Cortisol | luminescence enzyme immunoassay | Siemens Healthcare Diagnostics B.V., Breda, The Netherlands | 3.6–6.4% | 4.7–9.0% |  | 30 nmol/L | 220-650 (8 AM) |
| IL-6 | enzyme immunoassay | Pelikine Compact Elisa Kit, Sanquin, Amsterdam, The Netherlands | NA | NA |  | 1 pg/ml | <10 pg/ml (in healthy individuals) |
| **Sex hormones** | | | | | | |  |
| Estradiol | commercial radioimmunoassay (RIA) | Siemens Healthcare Diagnostics B.V., Breda, The Netherlands | 4.6–6.5% | 8.0–9.9% |  | 0.04 nmol/L |  |
| FSH | electro-chemiluminescence immunoassays (ECLIA) | Cobas E602, Roche diagnostics, Almere, The Netherlands | 1.2% at 9.7 E/L and 1.0% at 56.8 E/L |  | 2.5 at 9.7 E/L and 2.5% at 56.8 E/L | 1 IU/L |  |
| LH | ECLIA | Cobas E602, Roche diagnostics, Almere, The Netherlands | 1.7% at 4.7 E/L and 1.6% at 52.2 E/L |  | 1.6% at 4.7 IU/L and 1.6% at 52.2 E/L | 1 IU/L |  |
| Prolactin | solid-phase, two-site, time-resolved fluoroimmunometric assay | Delfia Prolactin, PerkinElmer, Turku, Finland | 4% at 5 µg/L and 6% at 24 µg/L | 5.5% at 4 µg/L and 7.2% at 50 µg/L |  | 1.0 µg/L |  |
| **Other hormones** | | | | | | |  |
| GH | time-resolved fluoroimmunoassay | Delfia, PerkinElmer, Turku, Finland | 6.4% at 3.4 mU/L and 1.8% at 20.1 mU/L | 10.9% at 3.0 mU/L and 7.7% at 21.7 mU/L |  | 0.1 mU/L (1 μg/L = 3.67 mU/L) |  |
| TSH | ECLIA | Cobas E602, Roche diagnostics, Almere, The Netherlands | 0.9% at 0.156 IU/L and 0.8% at 2.952 IU/L |  | 1.4% at 0.156 IU/L and 1.4% at 2.952 IU/L | 0.01 IU/L |  |
| **Peptides** | | | | | | |  |
| TGs | enzymatic colorimetric test | Cobas C702, Roche Diagnostics, Almere, The Netherlands | 0.9% at 1.32 mmol/L and 0.6% at 2.40 mmol/L |  | 2.0 at 1.39 mmol/L and 1.6% at 2.33 mmol/L | 0.1 mmol/L |  |
| FFAs | enzymatic method | NEFAC; Wako Chemicals, Neuss, Germany | 1% at 0.22 mmol/L and 1% at 0.93 mmol/L | 15% at 0.01 mmol/L and 4% at 0.48 mmol/L |  | 0.02 mmol/L | 0.14-0.44 (ULN is 0.44 mmol/L) |
| Glucagon | RIA | Linco Research, St. Charles, MO | 3–5% | 9–13% |  | 15 ng/L | 40-140 |
| Glucose | spectrophotometric method | Cobas C702, Roche diagnostics, Almere, The Netherlands | 0.8% at 5.3 mmol/L and 0.7% at 13.4 mmol/L |  | 1.3% at 5.3 mmol/L and 1.1% at 13.4 mmol/L | 0.1 mmol/L | 4.1-5.6 |
| Insulin | chemiluminescent immunometric assay | Siemens Healthcare Diagnostics B.V., Breda, The Netherlands | 6% at 47 pmol/L and 3% at 609 pmol/L | 4% at 91 pmol/L and 6% at 120 pmol/L |  | 15 pmol/L | 34-172 |
| PP | EURIA | RB316, Euro-Diagnostica AB, Malmö, Sweden | <3% | 10.3% at 23 pmol/L, 3.7% at 104 pmol/L and 5.5% at 208 pmol/L |  | 6 pmol/L | 0-100 |

HPA axis response: hypothalamic-pituitary-adrenal axis response; ACTH: adrenocorticotropic hormone; IL-6: interleukin 6; FSH: follicle stimulating hormone; LH: luteinizing hormone; GH: growth hormone; TSH: thyroid stimulating hormone; TGs: triglycerides; FFAs: free fatty acids; ULN: upper limit of normal; PP: pancreatic polypeptide.
