# Supplementary material for: Hormone, metabolic peptide, and nutrient levels in the earliest phases of rheumatoid arthritis—contribution of free fatty acids to an increased cardiovascular risk during very early disease
Source: Clin Rheumatol. 2016 Nov 2;36(2):269–78. doi: 10.1007/s10067-016-3456-x (PMC5290053; doi:10.1007/s10067-016-3456-x)
Supplement: Supplementary file 2 — (DOC 41 kb). [file 10067_2016_3456_MOESM2_ESM.doc]

**Supplementary Table 2. Characteristics of the study subjects in the validation cohort**

|  | RA (n=16) and UA (n=4) | Healthy control (n=20) | At risk for RA (n=32) | P |
| --- | --- | --- | --- | --- |
| Age (years) | 50 (39-59) | 50 (40-54) | 48 (36-55) | 0.83 |
| Female: n (%) | 15 (75) | 15 (75) | 21 (66) | 0.75 |
| BMI (kg/m^2^) | 25.7 (24.9-27.2) | 24.4 (22.3-27.6) | 24.8 (22.8-28.2) | 0.61 |
| Alcohol use: n (%) | 10 (50) | ND | 22 (69) | NA |
| Current smoker: n (%) | 10 (50) | ND | ND | NA |
| Pack years * | 20 (10-31) | ND | ND | NA |
| IgM-RF positive: n (%) | 15 (75) | 0 (0) | 20 (63) | NA |
| IgM-RF titer (kU/L) ** | 37 (33-75) | NA | 35 (20-79) | NA |
| ACPA positive: n (%) | 18 (90) | 0 (0) | 21 (65) | NA |
| ACPA titer (kAU/L) ** | 2320 (220-3422) | NA | 920 (150-2491) | NA |
| VAS GDA (mm) | 50 (24-59) | 0 (0-0) | 39 (7-62) | NA |
| ESR (mm/Hr) | 18 (9-34) | ND | 10 (3-20) | NA |
| CRP (mg/L) | 3.4 (1.2-9.3) | 1.1 (1.0-1.7) | 2.2 (1.0-4.7) | NA |
| DAS28 | 3.58 (3.08-4.56) | NA | 2.76 (1.39-3.30) | NA |
| NSAIDs: n (%) | 10 (50) | 0 (0) | 15 (33) | NA |
| Corticosteroids: n (%) | 0 (0) | 0 (0) | 0 (0) | NA |
| Methotrexate: n (%) | 10 (50) | 0 (0) | 0 (0) | NA |

Data presented as median (interquartile range) or number (percentage). P-values for Kruskal Wallis test for continuous variables and Pearson Chi-Square test for categorical variables. *P*<0.05 is considered statistically significant. RA: rheumatoid arthritis; UA: undifferentiated arthritis; BMI: body mass index; * only in smokers; IgM-RF: IgM rheumatoid factor; ** only in positive patients or individuals; ACPA: anti-citrullinated protein antibodies; VAS GDA: patient visual analogue scale (range 0-100 mm) global disease activity; ESR: erythrocyte sedimentation rate; CRP: C-reactive protein; HCQ: hydroxychloroquine; NA: not applicable; ND: not determined.
